# Perceptions of deaf subjects about communication in Primary Health Care^*^

**DOI:** 10.1590/1518-8345.2612.3127

**Published:** 2019-03-18

**Authors:** Alane Santana Santos, Arlindo José Freire Portes

**Affiliations:** 1 Instituto Nacional de Educação de Surdos, Divisão Médico Odontológica, Rio de Janeiro, RJ, Brazil.; 2 Universidade Estácio de Sá, Rio de Janeiro, RJ, Brazil.

**Keywords:** Accessibility, Primary Health Care, Communication Barriers, Communication, Hearing Loss, Deafness, Acessibilidade, Atenção Primária à Saúde, Barreiras de Comunicação, Comunicação, Perda Auditiva, Surdez, Accesibilidad, Atención Primaria a la Salud, Barreras de Comunicación, Comunicación, Pérdida Auditiva, Sordera

## Abstract

**Objective:**

to analyze the perceptions of deaf individuals about the communication process with health professionals of the state of Rio de Janeiro.

**Methods:**

cross-sectional observational study. Data were collected through the application of a questionnaire with quantitative and qualitative questions to 121 deaf adults. Objective responses were studied descriptively through frequency tables and analyzed by inferential statistics and logistic regression. The data from the open questions were analyzed through content analysis.

**Results:**

the lack of interpreters and the lack of use of the Brazilian Sign Language by professionals were perceived as the main communication barriers. In turn, the presence of companions who are listeners (73%) and the use of mime/gestures (68%) were among the strategies most used by the deaf. The majority of deaf people reported insecurity in consultations, and those who best understood their diagnosis and treatment were the bilingual deaf (p = 0.0347) and the deaf who used oral communication (p = 0.0056).

**Conclusion:**

communication with the professionals was facilitated when the deaf people had a companion or when they used mimics and gestures. Sign language was neglected, despite the fact that the provision of care to the deaf by professionals trained to use this language is guaranteed in the legislation.

## RESUMO

**Objetivo:**

analisar as percepções de indivíduos com surdez em relação ao processo comunicacional com profissionais de saúde da Atenção Básica do Estado do Rio de Janeiro.

**Métodos:**

estudo observacional transversal. A coleta de dados foi feita através da aplicação de questionário com perguntas quantitativas e qualitativas a 121 surdos adultos. As respostas objetivas foram estudadas descritivamente através de tabelas de frequência e analisadas por estatísticas inferenciais e de regressão logística. E, para os dados oriundos de questões abertas, foi realizada uma análise de conteúdo.

**Resultados:**

a falta de intérprete e a não utilização da Língua Brasileira de Sinais pelos profissionais foram percebidas como principais barreiras comunicacionais. Já a presença de acompanhante ouvinte (73%) e o uso de mímicas/gestos (68%) estão entre as estratégias mais utilizadas pelos surdos. A maioria dos surdos relatou insegurança após as consultas e os que melhor compreenderam seu diagnóstico e tratamento foram os surdos bilíngues (p=0,0347) e os oralizados (p=0,0056).

**Conclusão:**

a comunicação com os profissionais foi facilitada quando os surdos estavam com acompanhante ou quando utilizavam mímicas e gestos, sendo a língua de sinais negligenciada, apesar da legislação garantir aos surdos atendimento por profissionais capacitados para o uso desta.

## RESUMEN

**Objetivo:**

analizar las percepciones de individuos con sordera en relación al proceso comunicacional con profesionales de salud de la Atención Básica del Estado de Rio de Janeiro.

**Métodos:**

estudio observacional transversal. La recolección de datos fue hecha a través de la aplicación de un cuestionario con preguntas cuantitativas y cualitativas a 121 sordos adultos. Las respuestas objetivas fueron estudiadas descriptivamente a través de tablas de frecuencia y analizadas por estadísticas inferenciales y de regresión logística. Y, para los datos oriundos de preguntas abiertas, fue realizado un análisis de contenido.

**Resultados:**

la falta de intérprete y la no utilización de la Lengua Brasilera de Signos por los profesionales fueron percibidas como principales barreras comunicacionales. La presencia de acompañante oyente (73%) y el uso de mímicas/gestos (68%) están entre las estrategias más utilizadas por los sordos. La mayoría de los sordos relató inseguridad después de las consultas, y los que mejor comprendieron su diagnóstico y tratamiento fueron los sordos bilingües (p=0,0347) y los orales (p=0,0056).

**Conclusión:**

la comunicación con los profesionales fue facilitada cuando los sordos estaban con acompañante o cuando utilizaban mímicas y gestos, siendo la lengua de signos descuidada, a pesar de la legislación garantizar a los sordos atendimiento por profesionales capacitados para el uso de esta.

## Introduction

Comprehensive health care, with a view to autonomy of the subject, is one of the pillars of primary care. It is, therefore, imperative that communication between users and professionals occurs in a satisfactorily way in oder to preserve quality of care^1^ ^-^ ^2^.

The 2010 Brazilian demographic census indicated that 9.8 million people, or 5.1% of the Brazilian population, had hearing impairment^3^. It is known that the impairment caused by hearing loss, in terms of perception of sounds, can negatively impact people because of the importance of this ability to the development of communication, speech and language^4^.

It is important to highlight the difference between hearing impairment and deafness, according to the Brazilian legislation. Impairment is linked to hearing loss, while the deaf subject is perceived based on an identity, characterized by the use of sign language^5^.

In view of the need to maintain the quality of care, based on qualified listening, mutual conceptual understanding, interaction with the user and perception of his singularity, communication is fundamental in the work process of health professionals^1^ ^-^ ^2^.

In this scenario, when seeking health care, the main obstacles faced by deaf people involve the professionals’ lack of knowledge of sign language, and the lack of interpreters in the units^6^. It is important to note that such difficulties hamper the access of these subjects to health services. In the United Kingdom, a study revealed that the level of dissatisfaction with primary care physicians is higher among deaf patients than among listeners^7^.

In addition to the above, the lack of informative and accessible systems for the deaf increases their vulnerability to preventable diseases, as a result of lack of mechanisms that take into account the peculiarities of minority groups when disclosing health information^8^. In deaf communities in Nigeria, Brazil and the United States, a study pointed out that communication barriers inhibit the insertion of deaf people into health promotion programs and compromise their acquisition of knowledge^7^.

At the same time, communication barriers generate negative feelings and discourage deaf individuals to seek health units, because of fear of not being understood causes, making them to seek care only in case of illness. Therefore, it is fundamental that the professionals invest in strategies to facilitate the understanding and the reception of these subjects through effective communication^8^.

The present study sought to answer the following question: regarding the communication process established with health care professionals of the state of Rio de Janeiro, what are the perceptions of deaf individuals regarding the barriers and communication strategies used by them?

The objective of this research was to analyze the perceptions of deaf individuals about the process of communicating with primary health care professionals in the state of Rio de Janeiro.

## Methods

This is a cross-sectional, descriptive and analytical study with a mixed (qualitative and quantitative) approach^9^ ^-^ ^10^. Data were collected through the application of a questionnaire to deaf adults of the National Institute of Education of the Deaf (INES), located in Laranjeiras, Rio de Janeiro, and that assists students from Early Childhood Education to Higher Education. Data were collected in the period between 05/12/2016 and 03/22/2017.

For sample calculation, an expected response rate of 50% for any item of the questionnaire was considered, with an additional of 10% in case of possible losses, margin of error of 7%, and inferential statistics generated with a significance level of 5%, resulting in a sample of 121 deaf adults^10^.

As inclusion criteria, the age of the participant should be equal to or greater than 18 years and he should use of Brazilian Sign Language (LIBRAS) as a means of communication. The exclusion criteria were consultations performed in primary health care units of in Rio de Janeiro more than 5 years ago, or cognitive or neurological impairments that prevented the completion of the questionnaire.

Since no standardized and validated questionnaire for the Brazilian population of deaf people was found, a questionnaire published in a paper by Nascimento, Fortes and Kessler in 2015 was used for data collection. This questionnaire contained open and closed questions, and was adapted to fit the objectives of this study, after a pilot study with deaf individuals to detect possible nonconformities.

The instrument was applied in classrooms, in the break time, to groups of 5 to 10 students and with the collaboration of a LIBRAS interpreter/translator. After the presentation of the project and signing of the Informed Consent Term (ICT), the questions were translated one at a time, allowing time for all students to complete an item before passing to the next. At the end, all questionnaires were collected.

The data were organized into categories. Data from the objective questions were stored in a spreadsheet encoded using the Epi Info 7.2 software, and those from subjective questions were stored in an Excel 2010 worksheet.

Descriptive statistics included frequency tables for categorical variables, and means and standard deviation for quantitative variables. The association between variables was checked with the chi-square test and prevalence odds ratio were calculated. Multiple logistic regression was used for multivariate statistics^10^ ^-^ ^11^. Regarding the open questions, data were explored through content analysis based on Bardin^12^.

In order to identify the subjects and preserve their anonymity, the code UD/UT/Q/E followed by an Arabic numeral corresponding to the order of the questionnaires in the Excel 2010 worksheet was used, as for example: Q 1. The acronym UD is equivalent to Understanding of the diagnosis; UT means Understanding of the treatment, Q means Quality of care; and E means Thoughts and feelings of the deaf regarding their experiences during provision of care.

This study was submitted to the Ethics Committee of the Estácio de Sá University/UNESA/RJ, and was approved on November 11, 2016, under Opinion nº 1,818,244. In compliance with the ethical norms for research involving human beings, the application of the questionnaires was preceded by the signing of the Informed Consent Term (ICT).

## Results

The sample consisted of 121 adult deaf people, most of whom were male (58%) and had a mean age of 27 years (SD: 9.1 years). The participants attended high school and resided in the city of Rio de Janeiro, according to Table 1.

**Table 1:** Socio-demographic variables of the deaf participants, Rio de Janeiro, RJ, Brazil, 2016-2017

**Variables**	N*	**%** ^**†**^
**Sex**		
**Male**	**70**	**58**
**Female**	**51**	**42**
**Age**		
**18 - <28**	**82**	**68**
**28 - <38**	**20**	**17**
**38 - <48**	**16**	**13**
**48 - <58**	**2**	**2**
**58 - <68**	**1**	**1**
**Education**		
**Elementary School**	**39**	**32**
**High school**	**75**	**62**
**Higher education**	**7**	**6**
**Way of communication used** ^**‡**^		
**Sign language**	**110**	**91**
**Oral language**	**28**	**23**
**Bilingual**	**14**	**12**
**Residence**		
**Rio de Janeiro**	**92**	**76**
**Other municipalities**	**29**	**24**

*N - number; ^†^Percentage; ^‡^An individual may be present in more than one variable, thus the sum of percentages is greater than 100%

The absence of a mediator during the consultations, specifically a LIBRAS translator/interpreter or accompanying listeners, was responsible for 63% of the cases of withdrawn from seeking health units. Eighty-three percent of deaf people said they did not receive care in primary care unit from professionals who mastered LIBRAS.

The lack of LIBRAS interpreters, indicated by 85% of the deaf users, and the non-use of LIBRAS by professionals, indicated by 78% of the participants, were mentioned as the main communication barriers faced during health care.

Sixty-six percent of deaf people reported insecurity after consultations regarding the care provided by the medical professional, with respect to the diagnoses and treatments described. The safety the others felt was associated with the presence of a listener who communicated with the professional (72%), and only 13% of the participants reported feeling confident due to the communication strategies used during the provision of care.

As for the level of comprehension of the deaf individuals from the communication strategies used by the health professionals, 82% reported not understanding their diagnosis and 70% said they did not understand the guidelines for their treatment.

Sixty-one percent of deaf people who responded to the survey stated that health professionals did not understand them when they were alone. Thus, the presence of a companion who was listener (73%) and the use of mime/gestures (68%) were pointed out as the strategies most used by the deaf subjects to facilitate their communication during the consultations.

The use of written Portuguese (70%) and verbalization (54%) were pointed out by most of the subjects as strategies that most hinder communication between the deaf users and the professionals during the consultations.

Regarding the strategies that stimulated the independence of the deaf, 91% indicated the use of LIBRAS and 59% the presence of a LIBRAS interpreter/translator in the health units. As for privacy, the main strategy mentioned was use of LIBRAS, pointed out by 93% of the participants.

According to the Prevalence Odds Ratio, bilingual deaf individuals were approximately six-fold more likely to understand their diagnoses than those who were not. Similarly, deaf individuals who were able to verbalize were approximately twice as likely to understand their diagnosis as those who were not speakers. Deaf individuals who used signs were 79% less likely to fully understand their diagnosis (p = 0.0403) (Table 2).

The use of lip reading and verbalization as ways of communicating were also related to the deaf persons’ perception of their diagnosis. Therefore, deaf individuals who used lip reading and oral communication were 6.13-fold and 5.79-fold more likely to understand their diagnoses, respectively, when compared to individuals who did not use these methods of communication. It was also noted that the prevalence of deaf people who understood their diagnoses was 3.81-fold and 3.57-fold higher for those who used oral communication and lip reading, respectively (Table 2).

**Table 2:** Association between the variables and the level of understanding of the deaf people of their diagnosis, Rio de Janeiro, RJ, Brazil, 2016-2017

	**Variables**	PR* (95% CI) ^**§**^	**POR** ^**†**^ **(95% CI)** ^**§**^	**p** ^**‡**^
**Sex**	**Female**	**0.95 (0.44 -2.05)**	**0.94 (0.37 - 2.40)**	**1**
	**Male**	**1**	**1**	**1**
**Way of communication used**	**Bilingual**	**3.57 (1.77 -7.21)**	**6.13 (1.88 - 19.99)**	**0.0035** ^**||**^
	**Sign language**	**0.34 (0.16-0.74)**	**0.22 (0.06 - 0.80)**	**0.0403** ^**||**^
	**Verbalization**	**2.30 (1.10-4.80)**	**2.92 (1.09 - 7.82)**	**0.0567** ^**||**^
**Communication strategies used by the deaf**	**Oral communication**	**3.81 (1.84-7.89)**	**5.79 (2.15 - 15.55)**	**0.0005** ^**||**^
	**Mime and gestures**	**0.57 (0.27-1.21)**	**0.50 (0.19 - 1.28)**	**0.2243**
	**Written Portuguese**	**1.71 (0.78-3.74)**	**1.97 (0.70 - 5.49)**	**0.309**
	**LIBRAS** ^**¶**^	**0.27 (0.07-1.08)**	**0.22 (0.05 - 1.00)**	**0.0639**
	**Lip reading**	**3.57 (1.77-7.21)**	**6.13 (1.88 - 19.99)**	**0.0035** ^**||**^
	**LIBRAS** ^**¶**^ **interpreter**	**0.40 (0.06-2.71)**	**0.35 (0.04 - 2.80)**	**0.5109**
	**Figures**	**0**	**0**	**0.4353**
	**Drawings**	**0**	**0**	**0.3073**
	**Communication using the fingers**	**0**	**0**	**0.3652**
	**Companion who is a listener**	**0.66 (0.30-1.42)**	**0.59 (0.22 -1.58)**	**0.4272**

*PR - Prevalence Ratio; ^†^PCR - Prevalence Odds Ratio; ^‡^p - probability of significance; ^§^95% confidence interval; ^||^Statistically significant values, p < 0.05; ¶LIBRAS - Brazilian Sign Language

Regarding the knowledge about the treatment, the association with the way of communication used of the deaf person showed that bilingual individuals presented 5.33-fold greater chance to understand the health professional (p = 0.007). Likewise, if the deaf individual used lip reading and verbalization as ways of communicating, the chances of understanding treatment increased by five and three times, respectively. However, the deaf people who communicated through LIBRAS presented 67% less chance of understanding their treatment than deaf people who did not use it (p = 0.0538).

When they sought health care alone, the deaf users who used oral communication as a strategy to communicate increased their chances of being understood by health professionals by 2.93-fold in relation to those who did not use this communication strategy (p = 0.0221).

The multiple logistic analysis evaluated the influence of the variables age, sex, schooling (High school/elementary school, Higher education/elementary school) and way of communication used (verbalization, bilingual, sings) on the level of understanding of deaf people of their diagnosis and treatment, as well as the health professionals’ understanding of the information provided by the deaf user.

The variables affected the understanding of the diagnosis by the deaf user (p = 0.0091). When the associations were examined, it was observed that the way of communication of the individuals increased their chances of understanding the diagnosis. The chances of being understood were eight-fold higher in the bilingual compared to non-bilingual deaf (p = 0.0347), and among deaf people who used oral communication, the chance increased by 5.6-fold in relation to the ones who did not use oral communication (p = 0.0056).

Regarding the understanding the treatment guidelines, it was seen that bilingual users and individuals who used verbalization were more likely to understand, in the first case, 6.6 times more than non-bilingual users (p = 0.0556), and in the second, 3.28 times more than those who did not use verbalization (p = 0.0268).

It was observed that the level of education of deaf subjects influenced the understanding of information given by the health professional during the consultation. Individuals with high school were 3 (three)-fold more likely to be understood than those with only elementary school (p = 0.0125).

Table 3 summarizes the statistically significant variables (way of communication used and level of education) and their quantitative influence on the perception of deaf individuals of their diagnosis and treatment, as well as on the understanding of health professionals of the strategies they use.

**Table 3:** Multiple logistic regression, Rio de Janeiro, RJ, Brazil, 2016-2017

**Variables**		POR*	**95% CI** ^**†**^	**p** ^**‡**^
**Understanding of the diagnosis**				
**Bilingual**	**8.008**	**1.161**	**55.2361**	**0.0347** ^**§**^
**Verbalization**	**5.6536**	**1.6603**	**19.2514**	**0.0056** ^**§**^
**Understanding of the treatment**				
**Bilingual**	**6.605**	**0.9561**	**45.6275**	**0.0556** ^**§**^
**Verbalization**	**3.28**	**1.1463**	**9.3848**	**0.0268** ^**§**^
**Understanding by the professional**				
**Schooling (High School/ Elementary school)**	**3.3004**	**1.2926**	**8.4273**	**0.0125** ^**§**^

*PCR - Prevalence Odds Ratio; ^†^95% confidence interval; ^‡^p - probability of significance; ^§^Statistically significant values, p < 0.05

For qualitative analysis, the open questions were read, and the similarity between the answers, their frequency and the adequacy with the objectives of the study was examined. Then, the answers were grouped in Excel 2010 worksheets and divided into the following initial categories: understanding during the consultation; quality of care; and thoughts and feelings of the deaf regarding their experiences during provision of care^12^.

The first category, understanding during the consultation, was divided into two intermediate categories: understanding of the content addressed during the consultations and non-understanding, and these were divided into the final categories: written Portuguese language and absence of LIBRAS.

Most of the participants stated that the communication strategies used during the visits did not allow them to understand their diagnosis (82%) and their treatment (70%). Among the factors that hampered the understanding were the absence of LIBRAS and the use of written communication, as can be seen in the following reports: *I don’t understand because I have difficulty with writing and Portuguese* (UD 12); *I don’t understand the guidelines, because I only know how to communicate using LIBRAS* (UT 13).

Another factor to be considered is the attitude adopted by professionals during care: *Depending on the professional, I can understand the guidelines* (UD 3); *The communication strategies do not allow my understanding because the doctor does not have patience with deaf people* (UT 5); *I understand if the professional speaks slowly* (UD 6).

The second category, quality of care, was divided into two final categories: the use of LIBRAS and the presence of interpreters in the units. These were evaluated as measures to improve the quality of care, as it can be perceived in the following reports: *With an interpreter, it would be useful for the deaf people* (Q 5); *They need to learn LIBRAS or have interpreters in the units, to help the deaf to understand better* (Q 12); *To improve it is necessary an interpreter* (Q 40); *It would be nice if I had an interpreter everywhere* (Q 51).

With respect to the thoughts and feelings of the deaf users regarding the experiences during the consultations, 2 subcategories were created. The first one is related to the difficulties of communication faced and the second to the presence of companions in the consultation. The participants presented mixed feelings, among them indignation, anger and disappointment for not being understood or not understanding the health professional.

It was perceived in the responses that these feelings are due to both the difficulty to communicate and the lack of interest of the health professionals to improve this communication, who treat deaf users as if they were listeners rather than making an effort to treat them differently, and patiently seeking to facilitate communication. This is evident in the following statements: *I was not receive care, LIBRAS is necessary* (E 14); *We never understand anything, there is no communication (LIBRAS)* (E 20); *I cannot understand the doctor, because while I’m with my face in the device, he speaks behind me* (E 13); *If I go alone to the doctor, people talk normally, listeners speak as if they do not understand that I am deaf* (E 50).

The presence of a companion during the consultations was reported with frustration due to the lack of independence that translates and the embarrassment in relation to personal information that must be shared, as it can observed in the following speeches: *My mother always goes with me, it would be good to go alone* (E 3); *Mama going along is difficult* (E 5); *Whenever I go to the doctor, I need to go with my mother, but she does not know Libras, it’s difficult because I do not know what she talks to the doctor* (E 24); *Whenever I go to the health unit I need my daughter’s company, but she is not always willing to help me. It is difficult not to be able to communicate* (E 16); *My experience is bad because there is no interpreter and I have to go with my son or my mother to the doctor* (E 7).

However, in some cases the presence of the companion is seen as a relief because it enables communication and also the feeling of security, as described in the following statements: *My sister knows LIBRAS, she goes with me, I explain to her and she speaks with the doctor, because I cannot communicate with the doctor* (E 8); *I go to the doctor, you have no idea how difficult it is to understand people, my mother goes along to explain, it gets easier, so I use her as an interpreter* (E 9); *I always go to the health unit with my sister. If I need to go to the gynecologist I go with her because I feel afraid to go on my own* (E 18).

## Discussion

The rights of the deaf are guaranteed and regulated by law, which determines that the care in public health services must be provided by qualified professionals who know how to use LIBRAS or who can translate and interpret it^13^. However, it is noteworthy that, generally, the cultural identity of the deaf community is not taken into account; deaf people are devaluated as individuals and have their rights to equality in health care disrespected^14^.

In order to develop integral health care and promote social and structural changes, it is essential that the subjects be seen in their particularities. Understanding the reasons that pose a distance between them and the health units subsidize the remodeling and choice of strategies to receive these individuals.

The absence of caregivers and the lack of professional preparation were pointed as the main motivators for the deaf adults not to seek care in health services, according to studies conducted in Paraíba and Rio Grande do Sul^15^ ^-^ ^16^. In the present study, it was found that the absence of a mediator to facilitate communication with the professionals was a reason for the majority of deaf people to stop seeking care.

Considering the distinction between languages adopted by deaf people and listeners, it is possible that communicational difficulties exist between them. The obstacles most faced by the deaf participants in the present study involved the lack of interpreters in health facilities, the non-use of sign language by professionals, and the lack of patience on the part of professionals as well as their unpreparedness to assist such clientele.

In Brazil, sign language translators/interpreters have a regulated profession. They are responsible for interpreting and translating LIBRAS into Portuguese, as well as Portuguese language into LIBRAS. These professionals should promote communication between deaf people and hearing people and contribute to the accessibility of the deaf to public services^17^. Given the importance of communicational intermediation between deaf and hearing people, the absence of interpreters hampers the daily life of the deaf and encourages the adoption of other strategies that facilitate such process in the health units^3^ ^,^ ^17^.

In the face of communication barriers and lack of interpreters in health facilities, deaf people are forced to use someone as mediator, be it friends and/or family members. However, in many cases, the companions do not fully know LIBRAS, and this makes the intermediation enigmatic, generating anguish to the deaf subjects, for not knowing whether they were understood by the interlocutor and the health professionals^18^.

In spite of its importance to facilitate communication, the participation of a third party, compromises the privacy and the autonomy of the deaf people, and in some situations can cause embarrassment and omission of information because of exposure to shame^1^. This situation inhibits the deaf person to speak about her health, when they pass it to the other person the control over this information^18^.

Although studies indicate the presence of intermediary mediator as negative, in the present study, the presence of a mediator was highlighted as the strategy most used by the deaf to facilitate their communication with health professionals.

The choice for the presence of a companion can be associated with the absence of interpreters in the health units and lack of ability of the professionals to understand this clientele. Therefore, among the suggestions to increase the quality of care, the participants pointed out the need for the use of sign language by professionals and the relevance of the presence of LIBRAS interpreters in health units.

The communication barriers directly influence the perception that the deaf people have about the care provided, besides intensifying the dependence of these subjects on others. The difficulty to communicate and consequent deprivation of information implies a feeling of prejudice and discrimination of their disability, without considering their intellectual capacity and responsibility over their own health^18^.

Health care is directly linked to interpersonal relationships and requires communication skills for mutual understanding^19^. In the United States, a study involving 91 deaf adults revealed that communication difficulties led to low level of understanding of the subject regarding health guidelines and, consequently, feelings of fear, frustration and mistrust^20^.

Besides the doubts regarding their own health and the difficulty to understand the professional, the deaf also face the offer of very little information during the realization of procedures, which intensifies their insecurity and fear^18^. In a study carried out in São Luís-MA, it was noted that the lack of information for the deaf subjects was responsible for their difficulty to express their doubts and questions regarding their own health^18^.

Hence, the failure to embrace these clients generates negative feelings, that is, anguish, fear, insecurity and impatience, and, at the same time, pose a distance between professionals and the users^19^. The perception of the subjects is based on effective communication. However, after health care, deaf patients still misunderstand their diagnosis and treatment, a fact that ratifies the difficulty in communication between deaf people and health professionals^19^.

Likewise deaf subjects, health professionals recognize the need to overcome communication barriers. In a study carried out in Maranhão, the professionals identified the lack of training and the lack of resources to aid in communication as the main obstacles. In this scenario, the presence of a companion was pointed out as the main strategy used, and considered indispensable for the maintenance of an effective communication^21^.

It should be stressed that the main role of the deaf person should be maintained; the interaction with the professional should allow the individual to express his needs through strategies that ensure his independence and privacy^21^.

By guaranteeing the right to receive care from professionals trained in the use of LIBRAS, Decree nº 5,626, from December 22, 2005, stated the need to support for the training and improvement of professionals of the Unified Health System (SUS). However, such characteristics are not a reality for most deaf people^17^.

Attention to those who communicate in a different way requires that the professionals develop skills to use the most appropriate methods, preferably LIBRAS^1^. Assistance by professionals who know sign language enables communication without mediators and promotes the autonomy of the deaf^1^ ^,^ ^22^.

Legally recognized, LIBRAS characterizes the culture and identity of the deaf. Consequently, it is important that health professionals know LIBRAS; the lack of mastery of this language is a barrier to the interaction between the team and deaf individuals^23^.

The improvement of the quality of care for deaf users requires changes in the physical environment of the basic health units and in the training of professionals. Brazilian laws include LIBRAS as a compulsory topic for teacher training courses, speech therapy courses, and for all licentiate courses, being optional in other courses^22^.

The lack of content related to the care of deaf people during training may be one of the explanations for the difficulty of interaction between professionals and deaf users^22^. Therefore, it is important to emphasize the need for LIBRAS to be included in the curriculum of health professionals in order to promote the communication of deaf people with health professionals and to enable the integration of new entries in sign language^24^.

The investment in qualification does not ensure the training of health professionals interpreters or totally fluent in sign language, but enables the development of skills that allow effective communication with deaf users, with a view to social inclusion and respect for the rights of these subjects^17^.

Written Portuguese is frequently used by health professionals to communicate with deaf individuals. However, this is the second language of the deaf, and they often have difficulty understanding it fully^25^. The use of written Portuguese can embarrass the deaf individual and was described as the strategy that makes it more difficult to exchange information in the care.

The multiple logistic regression revealed that the difficulty in understanding written Portuguese is inversely proportional to the expansion of the level of education. Thus, deaf people who had finished high school were more likely to be understood by professionals than those who only finished the elementary school.

Our study evaluated deaf people who presented a considerable level of education, because they attended a reference institution. Studies with large samples among the deaf community, not linked to a reference institute, may show an even greater difficulty of these individuals to interact with primary health care professionals.

This study has as limitation the fact that the scenario is a national reference institution in education of deaf people. However, this made it possible to find a relevant sample of deaf people from the different municipalities of the state of Rio de Janeiro. The research took place in an environment that identifies all the subjects, made the sample homogeneous, and narrowed the variables of the study, without, therefore, allowing the crossing of variables, such as socio-demographic and cultural contexts of the subjects with those of deaf people from other places and realities.

After extensive bibliographic research, no other work was found in the Brazilian literature that addressed the barriers and communication strategies of deaf people in primary care in the SUS, following a quantitative and qualitative approach with more than 100 individuals surveyed.

Therefore, the recognition of the needs of this minority group contributes to the scientific progress in the area of education, based on a change in the training of professionals, as well as in the field of health, in which measures can be adopted with the purpose of enabling the care for deaf people, because it is based on the sensitization of these professionals that changes can be established.

## Conclusion

Despite the legal determination, it was clear that the deaf people are deprived of their rights because their first language, LIBRAS, is neglected. The present study pointed to the non-use of sign language by professionals and the absence of interpreters in health units as the main communication barriers faced by deaf subjects.

Communication barriers discourage the deaf to seek health units, influence the perception that these people develop of health care, and make them more dependent on mediators that facilitate the communication with professionals. Although favorable, in certain moments, the presence of a third party may generate uncertainties, fear, embarrassment, besides hindering the independence and autonomy of the deaf.

Regarding the direct interaction between users and professionals, it is evident that the knowledge and use of LIBRAS guarantee the respect for the privacy of the deaf. Therefore, it is essential to invest in the qualification of professionals and on their awareness in choosing communication strategies, taking into account the users’ needs, respecting their particularities, and the perception that the subjects hold a singular cultural identity.
